# Delayed Intracerebral Hemorrhage 15 Years After Indirect Revascularization in Moyamoya Disease: A Case Report and Review of the Literature

**DOI:** 10.3390/brainsci15101077

**Published:** 2025-10-04

**Authors:** Merih C. Yilmaz, Keramettin Aydin

**Affiliations:** Department of Neurosurgery, VM Medical Park Hospital, Samsun 55200, Turkey

**Keywords:** moyamoya disease, indirect revascularization, delayed intracerebral hemorrhage, multiple burr hole technique

## Abstract

**Background and Clinical Significance**: Moyamoya disease (MMD) is a progressive intracranial vasculopathy characterized by stenosis or occlusion of the terminal internal carotid arteries and the development of fragile collateral networks. It predisposes patients to ischemic and hemorrhagic strokes. Although both direct and indirect revascularization procedures are recommended to restore cerebral blood flow, recurrent cerebrovascular events may still occur, and delayed hemorrhage following revascularization is particularly uncommon. **Case Description**: We report the case of a 42-year-old woman who presented with seizure, syncope, and aphasia. Cranial computed tomography (CT) revealed a large left temporal–insular intraparenchymal hematoma with a midline shift. Computed tomography angiography (CTA) demonstrated bilateral internal carotid artery narrowing and collateral vessel proliferation, without aneurysm. Her history indicated a hemorrhagic stroke 15 years earlier, at which time MMD was diagnosed by magnetic resonance angiography (MRA) and managed with multiple burr hole surgeries. She remained free of cerebrovascular events until the current presentation. The patient underwent emergent hematoma evacuation, followed by intensive care management. Postoperatively, she demonstrated neurological improvement, though with residual motor aphasia and right-sided weakness, and was discharged for rehabilitation. **Conclusions**: This case underscores the rare occurrence of delayed intracerebral hemorrhage 15 years after indirect revascularization in MMD. Although revascularization surgery remains the standard therapeutic approach, this report highlights the importance of sustained long-term surveillance, strict risk factor management, and careful postoperative follow-up. The key point is that late hemorrhagic complications, though uncommon, must be considered in the long-term care of MMD patients following revascularization.

## 1. Introduction

Moyamoya disease (MMD) represents a cerebrovascular disorder that increases susceptibility to stroke due to progressive narrowing of the intracranial internal carotid arteries and their major proximal branches. Individuals who exhibit the distinctive moyamoya angiopathy in conjunction with additional underlying conditions are classified as having moyamoya syndrome [1]. MMD results in diminished cerebral perfusion, the development of collateral circulation, and a heightened susceptibility to transient ischemic attacks, ischemic strokes, and intracranial hemorrhage [1,2].

Epidemiological data indicate a higher incidence in people of Asian ancestry; however, cases are likewise observed in Western populations, including the Americas and Europe [3]. It was observed that the incidence peaked in the age group of 5 and 40 years [4,5]. MMD shows a female predominance, with an incidence about twice that in males. In the Japanese population, it is most frequently identified during childhood, and overall, the rate in Japan is estimated to be ten times greater than in European countries [4,5,6].

The etiology of MMD remains incompletely understood, but genetic factors, such as mutations in the ring finger protein 213 (RNF213) gene, have been implicated in disease susceptibility, particularly among East Asian patients [7].

Imaging modalities such as cranial computed tomography angiography (CTA), magnetic resonance angiography (MRA), and digital subtraction angiography (DSA) are employed for evaluation. The distinctive angiographic pattern of the collateral vessels, reminiscent of a “puff of smoke” in Japanese, led to the designation moyamoya.

Antiplatelet and anticoagulant agents are used in cases where surgery is not possible, but the primary treatment for MMD is revascularization surgery. Given its progressive nature and significant risk of ischemic and hemorrhagic complications, early recognition and appropriate management of MMD are critical to improving long-term neurological outcomes.

MMD, both ischemic and hemorrhagic strokes may occur following revascularization surgery. These complications are typically associated with certain etiological factors, such as hyperperfusion syndrome, hemodynamic alterations at the anastomosis site, or underlying vasculopathies. However, late-onset hemorrhagic stroke cases are reported to be quite rare in the literature. In this report, we present a rare case of delayed hemorrhagic stroke occurring long after revascularization and discuss it in the context of the existing literature.

## 2. Case Description

A 42-year-old female patient was admitted to the emergency department with complaints of seizure and syncope, initially presenting with aphasia. Upon admission, the patient’s blood pressure was recorded at 170/110 mmHg, with no prior history of hypertension. Laboratory investigations, including coagulation parameters, revealed no abnormalities.

Neurological examination showed impaired consciousness, with no anisocoria. She localized painful stimuli with the left extremities, whereas no movement was detected on the right side. There was no eye opening in response to verbal or painful stimuli (GKS 7).

**Figure 1:**
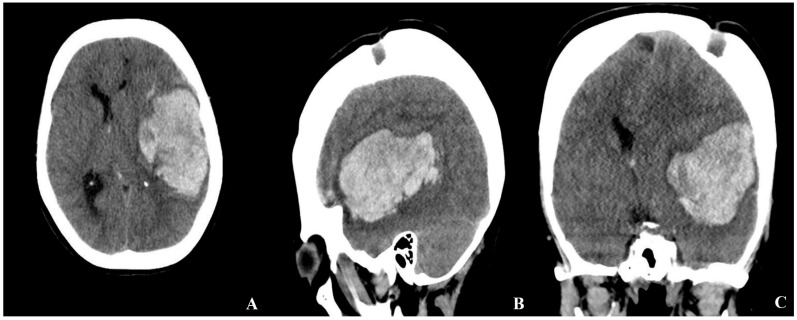
Initial brain CT obtained at the patient’s presentation to the emergency department ((**A**) axial, (**B**) sagittal, (**C**) coronal view). The scan demonstrates a large intraparenchymal hematoma in the left temporal and insular lobes, accompanied by a significant midline shift to the right and mass effect with compression of the adjacent ventricular structures. Multiple burr hole defects from the previous revascularization procedure are also visible.

An emergent brain CT demonstrated a 48 mm × 75 mm intraparenchymal hematoma in the left temporal and insular lobes, with a 16 mm midline shift to the right, as well as multiple burr hole defects (Figure 1).

**Figure 2:**
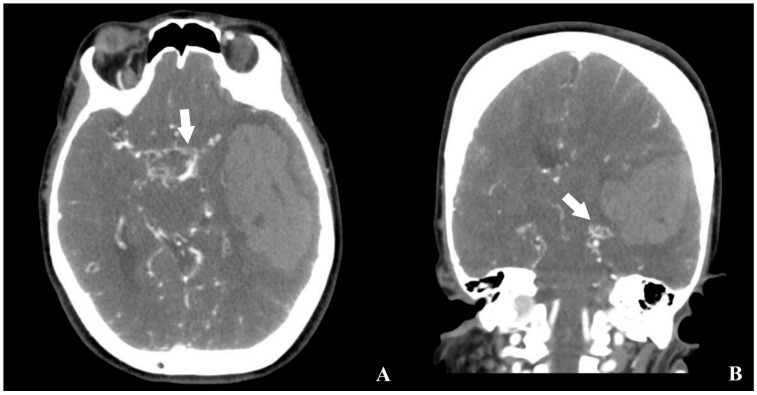
Preoperative brain CTA of the patient ((**A**) axial, (**B**) coronal, white arrow: revascularized region adjacent to the hemorrhage).

CT angiography revealed reduced calibers of the bilateral internal carotid arteries, along with prominent revascularization at the hematoma base and contralateral hemisphere. No aneurysms were identified (Figure 2).

**Figure 3:**
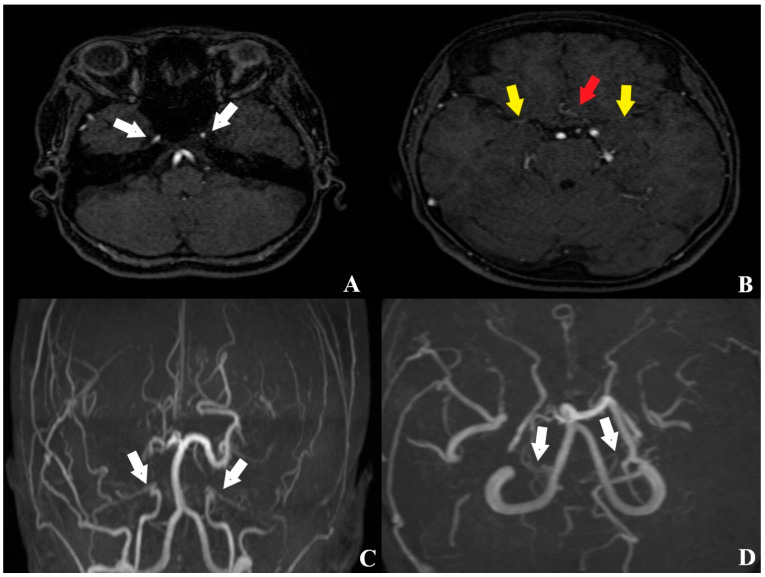
Pre-revascularization MR angiography obtained 15 years earlier ((**A**,**B**) axial planes; (**C**,**D**) 3D reconstructions). White arrow: decreased caliber of the internal carotid artery (ICA); yellow arrow: attenuated caliber of the middle cerebral artery (MCA); red arrow: reduced diameter of the anterior cerebral artery (ACA).

**Figure 4:**
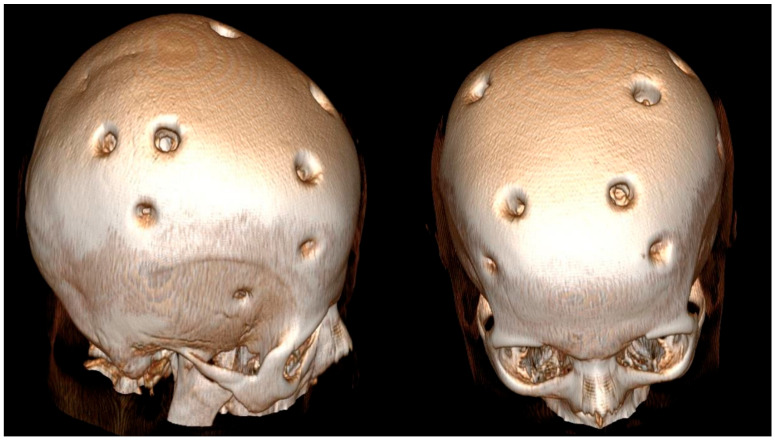
Three-dimensional brain CT reconstruction obtained 15 years after indirect revascularization, demonstrating the patient’s appearance following the multiple burr hole (MBH) procedure.

According to the history obtained from her husband, the patient had experienced a hemorrhagic stroke 15 years earlier, at which time MR angiography had confirmed the diagnosis of MMD, and she underwent multiple burr hole surgery (Figure 3 and Figure 4).

She had remained free of ischemic or hemorrhagic stroke for 15 years and had no comorbidities, including hypertension, except for Sjögren’s syndrome. Two years after the revascularization surgery performed 15 years ago, the patient was diagnosed with Sjögren’s syndrome following rheumatologic assessment for complaints of dry eyes and mouth. She was managed with symptomatic therapy, and her medical history indicated no evidence of disease progression.

The patient’s husband was informed, and written consent was obtained. The patient underwent a fronto-temporo-parietal skin incision, followed by the creation of an 8 cm × 6 cm craniotomy flap. Adhesions between the galea and dura from the previous revascularization were carefully dissected. Evacuation of the intraparenchymal hematoma exposed an area of atypical vascularization at its base, from which tissue samples were obtained for pathological analysis. The patient was subsequently transferred to the postoperative intensive care unit. In the postoperative period, the patient showed a favorable response to antihypertensive therapy with an angiotensin II blocker, diuretic, and calcium channel blocker, regaining consciousness and cooperative ability 34 h after surgery. Following a three-day stay in the intensive care unit, she was moved to the neurosurgery ward.

**Figure 5:**
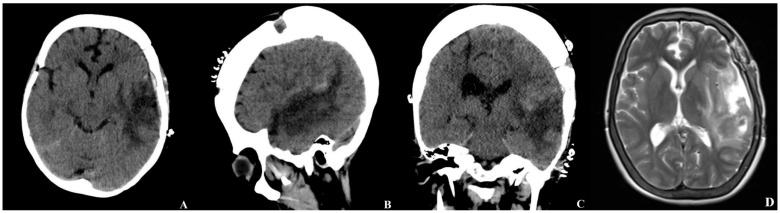
Brain CT and MRI obtained on the seventh postoperative day ((**A**) axial, (**B**) sagittal, (**C**) coronal view, (**D**) axial T2-weighted image). The postoperative CT demonstrates resolution of the previously observed midline shift and a marked decrease in perilesional cerebral edema. No new hemorrhage or ischemic changes are evident. The corresponding MRI sequences confirm a reduction in the mass effect, an improvement in ventricular compression, and postoperative changes consistent with hematoma evacuation.

During her ward stay, the patient developed diabetes insipidus, which was well-controlled with desmopressin therapy. Control CT and MRI obtained on postoperative day 7 demonstrated resolution of the midline shift and a decrease in cerebral edema compared with the preoperative findings (Figure 5). Histopathological evaluation revealed no evidence of vascular malformations.

At the time of discharge, the patient was maintained on antihypertensive therapy, including an angiotensin II receptor blocker, a diuretic, and a calcium channel blocker, with stable blood pressure. He was alert but exhibited motor aphasia, with right upper limb strength graded 2/5 and right lower limb strength 3/5 (mRS 3). He was discharged to continue physical therapy and rehabilitation.

## 3. Discussion

MMD impacts both children and adults, with clinical presentation ranging from cerebral ischemia and infarction to hemorrhagic lesions. Common manifestations include impaired cognition, migraine-type headaches, epileptic episodes, and motor disturbances such as abnormal contractions, muscular rigidity, and cramps [1,8,9,10]. In clinical series of MMD, the prevalence of ischemic stroke ranges from 50–75%, transient ischemic attacks from 50–75%, and intracranial hemorrhage from 10–40% [1]. In adult patients with MMD, enlargement and atypical branching of the anterior choroidal artery and/or posterior communicating artery serve as significant predictors of hemorrhagic episodes [11].

The etiology and mechanisms underlying MMD remain poorly understood. In the absence of an identifiable cause, it is considered an idiopathic disorder. Nevertheless, recent research has focused on clarifying potential genetic and immunological contributors.

MMD, previously regarded as a predominant genetic disorder, has also been linked to autoimmune mechanisms. A competitive endogenous RNA network centered on the long non-coding RNA MALAT1 was identified, involving 15 key mRNA targets. Immune profiling showed increased microvascular endothelial cells and reduced CD4+ memory and regulatory T cells, with MALAT1-associated gene expression correlating positively with endothelial changes and inversely with T-cell subsets. These findings indicate MALAT1 as a potential biomarker and therapeutic target in MMD [12].

MMD is most strongly linked to genetic alterations in RNF213, a founder mutation predominantly observed in East Asian populations. Emerging evidence demonstrates that RNF213 variants are also overrepresented in non-moyamoya intracranial vasculopathies, including large-artery atherosclerotic stroke and intracranial arterial stenosis or occlusion, particularly among East Asian individuals with early-onset or cryptogenic stroke [13]. Investigations of the RNF213 Arg4810Lys mutation have revealed that it disrupts angiogenic signaling, endothelial function, vascular remodeling, and immune regulation, effects worsened by stressors such as hypoxia and inflammation, and is implicated in MMD, intracranial stenosis, ischemic stroke, and atherosclerosis [7].

A ceRNA regulatory network was established to explore potential therapeutic targets in MMD, leading to the identification of AKT1, CLDN3, ISG20, and TGFB2 as critical hypoxia-immune–related genes implicated in disease mechanisms. These genes are believed to contribute to MMD pathogenesis through their involvement in processes such as epithelial–mesenchymal transition, angiogenic signaling, and cellular adhesion. Elucidating the function of hypoxia-immune genes in MMD not only provides insight into possible pathogenic mechanisms but also paves the way for novel diagnostic strategies and targeted therapeutic interventions [14].

Although the exact cause of MMD remains unclear, evidence indicates that immune dysregulation, alongside genetic predisposition, plays a key role. T and B lymphocytes, macrophages, and dendritic cells contribute to inflammation and vascular remodeling, promoting arterial stenosis and ischemic risk. Genetic and environmental factors influence immune activation, linking immune responses to disease progression. Targeting these pathways may offer therapeutic potential, but further research is needed to clarify mechanisms and optimize treatments [15].

Complement C3 levels are typically reduced in patients with MMD and show a further decline in the advanced stages as defined by Suzuki grading. Factors such as age, diastolic blood pressure, and circulating complement C3 levels have been associated with the progression of vasculopathy, supporting the hypothesis that the complement system may contribute to the pathogenesis of MMD [16].

Reports of MMD occurring in association with multiple sclerosis (MS) are rare, largely due to overlapping clinical features between the two conditions [17]. Although the characteristic angiographic findings enable the diagnosis of MMD, they do not exclude the possibility of concomitant central nervous system disorders. Without careful assessment of the clinical and neuroradiological profile, this coexistence may easily be overlooked. Therefore, in MMD patients presenting with atypical MRI lesions, cerebrospinal fluid analysis and spinal cord imaging are recommended to evaluate for potential accompanying pathologies [18].

In the differential diagnosis of MMD, it is important to consider conditions such as autoimmune disorders (e.g., Sjögren’s syndrome), meningitis, intracranial neoplasms, Down syndrome, Neurofibromatosis type 1, post-radiation cerebrovascular lesions, sickle cell disease, migraine, and atherosclerosis. Moreover, clinicians should remain aware that these conditions may coexist with MMD, potentially complicating the clinical and radiological assessment [19,20,21,22,23,24].

Digital subtraction angiography (DSA) remains the gold standard in diagnosing MMD. Owing to its superior spatial and temporal resolution, it is invaluable for evaluating stenosis or occlusion of the terminal internal cerebral artery (ICA) and the patency of collateral pathways. Additionally, DSA provides the most reliable means of visualizing collateral vessel development, which is essential for guiding therapeutic decisions and monitoring postoperative neoangiogenesis [25,26,27]. It is also a straightforward, efficacious, and safe approach when applied for the selective embolization of ruptured aneurysms arising within Moyamoya collateral vessels [28].

Computed tomography angiography (CTA) enables clear visualization of the circle of Willis as well as the anterior, middle, and posterior cerebral arteries and their main branches, offering a valuable diagnostic tool for detecting occlusive vascular pathology. Owing to its rapid acquisition and fast image reconstruction, CTA is often the preferred modality in emergency settings [29]. Computed tomography perfusion (CTP) is commonly utilized as the primary modality for evaluating postoperative cerebral hemodynamic alterations, owing to its rapid acquisition and superior spatial resolution [30].

Magnetic resonance angiography (MRA) provides a noninvasive and radiation-free alternative to DSA and CTA for evaluating bypass patency and allows assessment of arterial diameter to predict the formation of surgical collaterals [31]. Among available methods, 3D time-of-flight (TOF) MRA is the most frequently employed for cerebral vascular imaging, owing to its high spatial resolution, favorable signal-to-noise ratio, and capability of generating very thin slices. Nonetheless, TOF-MRA has demonstrated limitations compared with CTA, including reduced accuracy in visualizing trephination bypass segments and the tendency to overestimate focal pseudo-occlusive changes in this region [32]. Intracranial vessel wall imaging (IVWI) serves as a complementary technique to conventional MRA and holds significant promise for the morphological evaluation of revascularization in MMD. High-resolution MRI–based IVWI is valuable in distinguishing MMD from intracranial atherosclerotic stenosis [33,34]. In addition, preoperative assessments suggest that IVWI may act as a predictive marker for postoperative stroke within the affected vascular territory following revascularization [35]. The “Ivy sign,” characterized by leptomeningeal hyperintensity on fluid-attenuated inversion recovery MRI (FLAIR-MRI), is considered a distinctive radiological marker of MMD, reflecting sluggish flow within dilated pial vessels supplying leptomeningeal collaterals and regions of compromised perfusion [36]. Clinical studies have demonstrated that regression of the “ivy sign” on postoperative FLAIR-MRI correlates with improved cerebral hemodynamics and symptomatic relief, whereas the emergence of a new “ivy sign” may serve as an early indicator of postoperative cerebral hyperperfusion syndrome [37].

Blood oxygen level–dependent functional MRI (BOLD-fMRI) is a neuroimaging modality that exploits deoxyhemoglobin within cerebral vessels as an intrinsic contrast mechanism to generate functional activation maps. It is extensively applied to evaluate cerebrovascular reserve, alterations in neurovascular coupling, and to determine the effectiveness of surgical revascularization [38,39,40]. BOLD-fMRI also shows promise as a standardized tool for both pre- and postoperative assessment of MMD patients, particularly in the pediatric population. Nonetheless, enhancing the reliability and reproducibility of BOLD imaging remains a significant challenge in clinical practice [29].

Single-photon emission computed tomography (SPECT) is regarded as the gold standard for evaluating cerebral perfusion [41]. Reduced cerebrovascular reserve, detected either at baseline or following acetazolamide stimulation in postoperative MMD patients, is associated with an unfavorable prognosis, including ongoing neurological deficits and recurrent ischemic events [42]. Furthermore, alterations in cerebral blood flow measured by SPECT have been linked to cognitive recovery in both adult and pediatric populations with MMD [43].

Positron emission tomography (PET) is regarded as the reference functional imaging modality for evaluating metabolic processes associated with vascular function in MMD patients following bypass surgery [29]. Literature evidence suggests that PET provides more precise assessments for carefully selected patients who are most likely to derive benefit from surgical intervention [44,45,46]. Nonetheless, its widespread clinical application is constrained by limited availability, high cost, and prolonged acquisition times [29].

A novel scoring system, the MRI-Based Assessment of Risk for Stroke in Moyamoya Angiopathy (MARS-MMA), was created to evaluate hemodynamic status in patients with Moyamoya prior to revascularization and was compared with DSA and PET imaging. The MARS-MMA score demonstrated a strong correlation with cerebral perfusion reserve. This fully MRI-based scoring system represents a potentially valuable tool for predicting stroke risk in MMD patients [47].

Suzuki and Takaku proposed a grading system for MMD based on angiographic findings, outlining the progressive stages of vascular changes. In Grade I, there is initial narrowing at the internal carotid artery (ICA) apex. Grade II is characterized by the emergence of moyamoya collateral networks, while Grade III reflects further stenosis of the ICA accompanied by intensification of these abnormal collateral vessels. In Grade IV, collateral circulation begins to develop from the external carotid artery (ECA). Grade V demonstrates prominent ECA collateral formation alongside regression of moyamoya-related vessels. Finally, Grade VI represents complete occlusion of the ICA with the disappearance of moyamoya collaterals. This staging system provides a structured framework for evaluating disease progression and guiding clinical management [48].

In the surgical management of MMD, the ECA commonly serves as the donor source for augmenting blood supply to the ischemic cerebral hemisphere. Revascularization can be achieved through either direct or indirect approaches. In direct bypass, a branch of the ECA is surgically anastomosed to a cortical artery, thereby providing immediate blood flow augmentation. In contrast, indirect techniques involve placing vascularized tissue supplied by the ECA in contact with the brain surface, stimulating angiogenesis and the subsequent penetration of new vessels into the underlying cerebral cortex [1].

Another investigation reported that the combined revascularization strategy offers the advantage of direct anastomosis, ensuring an immediate increase in cerebral blood flow, while simultaneously promoting the gradual development of indirect collateral circulation. Nonetheless, it has been proposed that patients presenting with stenosis of the ICA or middle cerebral artery (MCA), but without complete occlusion, may be more suitable candidates for indirect bypass, as an immediate augmentation of blood supply is not essential in this subgroup [49].

A review of the literature highlights multiple studies comparing the outcomes of direct and indirect revascularization techniques. A systematic analysis demonstrated that postoperative stroke incidence following direct or combined bypass is comparable to that observed after indirect procedures [50].

In a cohort study involving 559 patients with MMD who underwent direct, indirect, or combined revascularization, a prior history of intracranial hemorrhage was identified as a predictor of postoperative hemorrhagic stroke. Moreover, comorbid conditions such as hypertension and diabetes were recognized as risk factors for postoperative ischemic stroke [51,52,53].

The periventricular anastomosis theory has been proposed to explain the hemorrhagic mechanism in MMD. Although direct bypass reduces the risk of intracranial bleeding, a modified approach termed “personalized targeting bypass” aims specifically at hemorrhage prevention by using multimodal imaging to select the target vessel at the site of medullary artery extension from the periventricular anastomosis. In a series of eight patients, marked postoperative stenosis of the targeted anastomosis was achieved, and no recurrent hemorrhage was observed during follow-up [54]. Moreover, five days of postoperative bed rest following direct bypass markedly lowered the risk of intracerebral hemorrhage and neurological decline [55].

Revascularization surgery has been shown to decrease the formation of moyamoya collateral vessels, thereby more effectively reducing the risk of hemorrhage compared with conservative management. In adult patients with MMD, direct bypass is particularly crucial for preventing rebleeding, as indirect techniques demonstrate lower efficacy than in pediatric cases [56]. Nevertheless, certain reports have suggested the presence of hemorrhagic complications following direct or indirect revascularization procedures in both the early and late postoperative periods. In addition, although these techniques have demonstrated efficacy in decreasing ischemic events related to MMD, they do not appear to increase the incidence of postoperative hemorrhage, and additional research is warranted to clarify the potential risk factors [57].

In a study evaluating the impact of hypertension on recurrent hemorrhage and long-term survival in patients with hemorrhagic MMD treated with indirect revascularization, strict blood pressure management was shown to markedly reduce hematoma recurrence but did not affect overall survival. These findings suggest that blood pressure optimization alone is insufficient in the management of hypertensive patients with hemorrhagic MMD [58,59].

The multiple burr holes (MBH) approach provides several benefits compared to craniotomy, such as reduced operative duration, lower intraoperative blood loss, fewer postoperative adverse events, and similar perfusion as well as functional results. In addition, MBH represents a safe and effective option for anterior cerebral artery revascularization in pediatric MMD patients [60]. Another investigation demonstrated that the MBH method could lower the likelihood of recurrent cerebrovascular events and decrease the incidence of pseudomeningocele [61]. The adjunctive use of erythropoietin (EPO) with the MBH procedure has been reported to decrease the incidence of long-term cerebrovascular events in MMS patients following either hemorrhagic or ischemic stroke [62].

Although reports of delayed intracerebral hemorrhage following direct or indirect revascularization are uncommon [63,64,65], our case involved hemorrhage occurring 15 years after indirect revascularization. This rare occurrence is examined in the context of existing literature.

In limitation, this study is constrained by its single-case design, which precludes generalization to broader patient populations. Genetic analysis, particularly regarding RNF213 or other susceptibility loci, was not performed, limiting insights into potential hereditary contributions. Advanced hemodynamic and functional imaging modalities, such as SPECT, PET, or BOLD-fMRI, were not obtained because the patient required urgent intervention, and only emergent CT angiography was performed. Detailed longitudinal neurocognitive and functional assessments were also unavailable, restricting evaluation of recovery beyond motor outcomes. Finally, given the rarity of delayed hemorrhage after indirect revascularization, further accumulation of similar cases and multicenter analyses are necessary to better understand risk factors and optimize long-term management strategies in MMD.

## 4. Conclusions

MMD is an intracerebral vasculopathy that may present with either hemorrhagic or ischemic stroke. Although genetic and immunological factors have been implicated in its etiology, the precise underlying cause remains unknown. Therapeutic options with conservative management are limited, and both direct and indirect revascularization procedures are generally recommended. Nevertheless, recurrent ischemic or hemorrhagic events may occur following revascularization surgery. In this report, we present a rare case of hemorrhagic stroke occurring 15 years after indirect revascularization and discuss the diagnosis, etiology, and postoperative complications of MMD in light of the current literature. Importantly, long-term follow-up should include monitoring of collateral vessel remodeling, the hemodynamic reserve, episodic hypertension, and the presence of coexisting autoimmune conditions such as Sjögren’s syndrome, as these factors may influence the risk of delayed hemorrhage and guide optimal patient management.

## Data Availability

The data presented in this study are available on request from the corresponding author due to the inclusion of personal radiological information in the case report.

## Abbreviations

The following abbreviations are used in this manuscript:

MMD	Moyamoya disease
CTA	Computed tomography angiography
MRA	Magnetic resonance angiography
DSA	Digital subtraction angiography
GKS	Glasgow coma scale
mRS	Modified Rankin scale
TOF	Time of flight
FLAİR	Fluid-attenuated inversion recovery
IVWI	Intracranial vessel wall imaging
CTP	Computed tomography perfusion
BOLD-fMRI	Blood oxygen level–dependent functional MRI
SPECT	Single-photon emission computed tomography
PET	Positron emission tomography
MARS-MMA	MRI-Based Assessment of Risk for Stroke in Moyamoya Angiopathy
MBHs	Multiple burr holes
